# Supplementary material for: Analysis of PPARγ Signaling Activity in Psoriasis
Source: Int J Mol Sci. 2021 Aug 10;22(16):8603. doi: 10.3390/ijms22168603 (PMC8395241; doi:10.3390/ijms22168603)
Supplement: Supplementary file 1 [file ijms-22-08603-s001.zip › Supplemental materials_Analysis of PPARg signaling activity in psoriasis/Pathway models/Models images and html files/Interleukin-17 and interleukin-22 signaling in psoriasis/142691.html]

chemotaxis


# Cell Process chemotaxis

|  |  |
| --- | --- |
| URN | urn:agi-gocellproc:0006935 |
| Total Entities | 91 |
| Connectivity | 4641 |
| Name | chemotaxis |

---

|  |  |
| --- | --- |
| ChildConcepts | cell tethering |
|  | cell rolling |
|  | immune cell chemotaxis |
|  | monocyte chemotaxis |
|  | mast cell chemotaxis |
|  | basophil chemotaxis |
|  | neutrophil chemotaxis |
|  | eosinophil chemotaxis |
|  | macrophage chemotaxis |
|  | lymphocyte chemotaxis |
|  | leukocyte tethering / rolling |
|  | positive chemotaxis |
|  | negative chemotaxis |
|  | dendritic cell chemotaxis |
|  | natural killer cell chemotaxis |
|  | B-cell chemotaxis |
|  | endothelial cell chemotaxis |
|  | granulocyte chemotaxis |
|  | phagocyte chemotaxis |
|  | microglia chemotaxis |
|  | T-cell chemotaxis |
|  | fibroblast chemotaxis |
|  | adipose macrophage chemotaxis |
|  | stem cell chemotaxis |
|  | myeloid cell chemotaxis |
|  | mast cell precursor chemotaxis |
|  | regulatory T-cell chemotaxis |
|  | Kuppfer cell chemotaxis |
|  | PBMC chemotaxis |
|  | capillary endothelial cell chemotaxis |
|  | M1 macrophage chemotaxis |
|  | blood platelet chemotaxis |
|  | hematopoietic cell chemotaxis |
|  | bone marrow stromal cell chemotaxis |
|  | mononuclear cell chemotaxis |
|  | umbilical vein endothelial cell chemotaxis |
|  | Th1 cell chemotaxis |
|  | decidual natural killer cell chemotaxis |
|  | Th2 cell chemotaxis |
|  | helper T-cell chemotaxis |
|  | CD8+ T-cell chemotaxis |
|  | effector T-cell chemotaxis |
|  | plasmacytoid dendritic cell chemotaxis |
|  | memory B-cell chemotaxis |
|  | bone marrow stem cell chemotaxis |
|  | antigen-presenting cell chemotaxis |
|  | B-1a B-cell chemotaxis |
|  | Langerhans cell chemotaxis |
|  | myeloid progenitor cell chemotaxis |
|  | mononuclear phagocyte chemotaxis |
|  | myofibroblast chemotaxis |
|  | CD4+ T-cell chemotaxis |
|  | colony forming cell chemotaxis |
|  | sinusoidal cell chemotaxis |
|  | cytotoxic T-cell chemotaxis |
|  | M2 macrophage chemotaxis |
|  | lung fibroblast chemotaxis |
|  | neutrophil precursor chemotaxis |
|  | haematopoietic stem cell chemotaxis |
|  | memory T-cell chemotaxis |
|  | alveolar macrophage chemotaxis |
|  | activated natural killer cell chemotaxis |
|  | thymocyte chemotaxis |
|  | fibrocyte chemotaxis |
|  | promonocyte chemotaxis |
|  | kidney mesangial cell chemotaxis |
|  | tumor infiltrating macrophage chemotaxis |
|  | conventional dendritic cell chemotaxis |
|  | Th22 cell chemotaxis |
|  | myeloid derived suppressor cell chemotaxis |
|  | Th17 cell chemotaxis |
|  | activated T-cell chemotaxis |
|  | killer cell chemotaxis |
|  | cytokine-induced killer cell chemotaxis |
|  | peritoneal macrophage chemotaxis |
|  | bone marrow derived mesenchymal stem cell chemotaxis |
|  | Vg9/Vd2 T-cell chemotaxis |
|  | immune system cell chemotaxis |
|  | dermal microvascular endothelial cell chemotaxis |
|  | T-lymphoblast chemotaxis |
|  | bone marrow derived mast cell chemotaxis |
|  | bone marrow derived dendritic cell chemotaxis |
|  | inflammatory monocyte chemotaxis |
|  | megakaryocyte chemotaxis |
|  | null cell chemotaxis |
|  | foam cell chemotaxis |
|  | marginal zone B-cell chemotaxis |
|  | plasma cell chemotaxis |
|  | natural killer T-cell chemotaxis |
|  | bone marrow macrophage chemotaxis |
|  | B-lymphoid precursor cell chemotaxis |

---

|  |  |
| --- | --- |
| Pathway | Leukocyte Adhesion to Endothelial Cell |
|  | CXC Chemokine Receptor Signaling |
|  | CC Chemokine Receptor Signaling |
|  | Histamine Effects in Inflammation |
|  | Basophil Activation |
|  | Eosinophil Activation |
|  | Mast-Cell Activation without Degranulation through TNFSF8 Signaling |
|  | Mast-Cell Activation via IgE Signaling |
|  | Mast-Cell Activation without Degranulation through CRHR1 Signaling |
|  | Mast-Cell Activation without Degranulation |
|  | Mast-Cell Activation without Degranulation through IL33/IL1RL1 Signaling |
|  | Mast-Cell Activation without Degranulation through KIT/F2R Signaling |
|  | Mast-Cells De Novo Synthesized Mediators via IgE Independent Signaling |
|  | Mast-Cell Activation without Degranulation through IL1R1 and TLR Signaling |
|  | Macrophage M1 Lineage |
|  | anti-Inflammatory Function of Macrophage M2 Lineage |
|  | Positive Acute Phase Proteins Synthesis |
|  | Scavenger Receptor OLR1 in Inflammation Related Endothelial Dysfunction |
|  | IL17 Signaling in Psoriasis |
|  | Toll-like Receptors in beta-Cell |
|  | Thymic Follicular Hyperplasia |
|  | Eosinophil Survival in Asthma |
|  | Il17 Signaling Related Neutrophilia in Asthma |
|  | IL33 Signaling Related Eosinophilia in Asthma |
|  | IL17F Signaling in Bronchial Epithelial Cell in Asthma |
|  | Basophil Activation in Asthma |
|  | Mast-Cell Related Chemokines Signaling in Asthma |
|  | Antigen-Presenting Cell Role in Asthma |
|  | Th17-Cell Differentiation in Asthma |
|  | WHIM Syndrome |
|  | CCR5 Signaling in Macrophages |
|  | IL36 Receptor Antagonist (IL1F5) Deficiency (DITRA) |
|  | Cytokines Trigger Otitis Media |
|  | CXCR4 -> ARRB1 Signaling |
|  | TGFBR3 -> ARRB2 Signaling |
|  | PPARG inhibits phenotypes assotiated with psoriasis |
|  | efects selected |
|  | Neighbors of chemotaxis |
|  | 2\_Interleukin-17 and interleukin-22 signaling in psoriasis |
|  | 1\_Th2 cell response in asthma |
|  | 2\_1\_Eosinophilia and neutrophilia in asthma: eosinophil migration and activation |
|  | Interleukin-17 and interleukin-22 signaling in psoriasis |

---

|  |  |
| --- | --- |
| MedScan ID | 4005109 |

---

|  |  |
| --- | --- |
| Alias | chemotactic |
|  | response to chemoattractant |
|  | chemotaxis |
|  | chemo-taxis |
|  | responses to chemoattractant |
|  | chemoattractant responses |
|  | chemoattractant response |

---

|  |  |
| --- | --- |
| GO ID | 0006935 |

---
